# Guided immediate implant with and without using a mixture of autogenous and xeno bone grafts in the dental esthetic zone. A randomized clinical trial

**DOI:** 10.1186/s13104-023-06612-8

**Published:** 2023-11-13

**Authors:** Sherine Osama El Ebiary, Mohammed Atef, Medhat Sameh Abdelaziz, Mohammed Khashaba

**Affiliations:** 1 Department of Prosthodontics, Faculty of Oral and Dental Medicine, Cairo University, Giza, Egypt; 2 Department of Oral and Maxillofacial Surgery, Faculty of Oral and Dental Medicine, Cairo University, Giza, Egypt; 3 Department of Prosthodontics, Faculty of Oral and Dental Medicine, Future University in Egypt, Fifth Settlement, End of 90 street, New Cairo, Cairo, Egypt

**Keywords:** Esthetics, Implants, Immediate implants, Surgical guides, CAD-CAM, Temporary prosthesis, Immediate loading

## Abstract

**Purpose:**

This in vivo study aims to assess the pink esthetic score in the anterior maxilla after computer-guided immediate implant installation and fully digital immediate temporalization with and without grafting the jumping gap with a mixture of 1:1 autogenous and xenograft particulates.

**Materials and methods:**

Twenty-four patients with non-restorable upper anterior teeth in the aesthetic zone have undergone a traumatic extraction for the non-restorable tooth followed by immediate implant placement using a 3D-printed surgical guide according to prosthetically driven implant placement. The patients were divided into two groups. The study group received the dental implant after grafting the jumping gap with 1:1 autogenous and xenograft particulates, while the control group received the dental implant without grafting the jumping gap. Each patient received a digitally fabricated, immediate, nonfunctional temporary prosthesis. The esthetic outcome was compared between the two groups using the pink esthetic score at implant insertion and after 6 months of follow-up. Statistical comparisons were carried out between the studied groups using the Mann-Whitney U test.

**Results:**

Immediately postoperatively, there was no statistically significant difference between the median PES in the two groups (P-value = 0.746). After six months, the study group showed a statistically significantly higher median PES than the control group (P-value = 0.048).

**Conclusions:**

Grafting the jumping distance in the immediate implant protocol helps achieve a better esthetic outcome.

**Clinical relevance:**

The use of immediate guided implant placement along with grafting the jumping gap followed by immediate digital temporalization guarantees a better esthetic outcome while preserving time, cost, and the number of clinical visits.

**Trial registration:**

The study was registered on clinicaltrials.gov with registration number NCT04096209. (19/9/2019)

## Introduction

One of the main goals that modern dentistry strives to achieve is the successful and predictable restoration of missing teeth. Many materials and treatment modalities have been introduced for tooth replacement in the esthetic zone. The introduction of dental implants has brought this goal closer, leading to a revolutionary era for successful treatment [1–4].

Immediate implant placement following tooth extraction has shown comparable survival rates to implants placed in healed alveolar ridges [5]. One of the common esthetic complications with immediate implants is mid-buccal gingival recession and the appearance of implant metallic shadow, especially with a thin gingival biotype [6]. To minimize these undesired esthetic complications, several techniques have been suggested, such as the application of connective tissue grafts, grafting the jumping gap with bone grafts, and the use of immediate temporalization [6].

Different techniques have been used for grafting the jumping gap such as Autograft based, Allograft-based bone which could be used alone or in combination with other materials for example: freeze-dried bone, Xenograft such as bovine bone, and Ceramic-based bone graft substitutes that include calcium phosphate, calcium sulfate, and bio-glass used alone or in combination [7–9].

Bovine bone substitutes counteract many of the dimensional changes that occur in the post-extraction sockets. Bovine bone grafts act as an ideal barrier material, which in turn preserves the ridge width and volume and supports the soft tissue, preventing it from collapsing [10]. A study conducted by Bianchi S reported that the use of xenografts showed important cellular activity that improves the osteoconductive properties in bone regeneration [8].

The bone formation around an implant is an important factor for a better esthetic outcome; on the contrary, any bone loss means the loss of soft tissue, which in turn affects the ideal implant aesthetic result [2, 11]. Many factors affect the outcome of immediate implant placement. As such, the surgical approach, whether guided flapless surgery or full flap surgery, the implant type, the type of bone substitute, and the crown design and technique of fabrication [6, 10].

With the advancement of computer-aided design and computer-aided manufacturing (CAD-CAM) technology, there are different approaches for implant prosthetics’ digital workflow [12–14]. Recent digital technology allowed the virtual extraction of unrestorable teeth, the design of a restoration with a perfect emergence profile that preserves soft tissue contour, and finally the 3D printing of the interim restoration [11, 15–17].

The CAD-CAM guided templates have many applications in dentistry such as implant placement and endodontic access preparations, the guided implant osteotomy presents an accurate protocol for implant placement in the preplanned position with less surgical time and few post-surgical complications. The digitally designed surgical guides transfer the virtually planned implant angulation, width and length through the use of 3D printed transparent template [18, 19].

Many debates with different opinions have existed regarding grafting or leaving the buccal jumping gap empty. Therefore, the primary purpose of this in vivo study was to evaluate the pink esthetic score in the maxillary esthetic zone after computer-guided immediate implant installation and fully digital immediate temporalization with and without grafting the jumping gap with a mixture of 1:1 autogenous and xenograft particulates.

## Materials and methods

The present parallel randomized control study was conducted following the ethical principles of the Helsinki Declaration for research on human subjects and was approved by the research ethics committee, faculty of dentistry, Cairo University (19)-(7)-(4). The study was registered on clinicaltrials.gov with registration number NCT04096209. It was first registered on September 19, 2019. Twenty-four patients with non-restorable upper anterior teeth in the aesthetic zone, indicated for extraction followed by immediate implant installation therapy, ages ranging from 20 to 50, were recruited from the outpatient clinic of the Oral and Maxillofacial Department, Cairo University.

The inclusion criteria were patients with non-restorable single bounded maxillary incisors or canines that have completely intact labial plate and interproximal bone levels and are indicated for implant placement. All patients have adequate bone height apical to the failing tooth. All patients were free from any debilitating diseases such as bone diseases, diabetes mellitus, or any other diseases that affect dental implant osteointegration. Informed consent was obtained from all the participants in the study.

The participants were randomly divided into two groups using block randomization with stratification (block size 4) using a formula on Microsoft Excel software.

The sample size was calculated using G Power software and based on a previous study by Diana C. et al. [20] The total sample size was twelve participants per group, with a study power of 80% at an α error probability of 0.05.

### Designing surgical guides and temporary restorations

A Cone-Beam Computed Tomography x-ray (PaX-i3D Green; VATECH), with parameters of 120 kVp,37.07 MAs, and a 0.25 mm voxel size, was performed for each patient to obtain Digital Imaging and Communication in Medicine (DICOM) file. Intraoral scanning for both working and opposing arches, along with digital bite registration, was carried out using the (MEDIT i600; MEDIT Corp.) to create a standard tessellation language (STL) file.

**Fig. 1:**
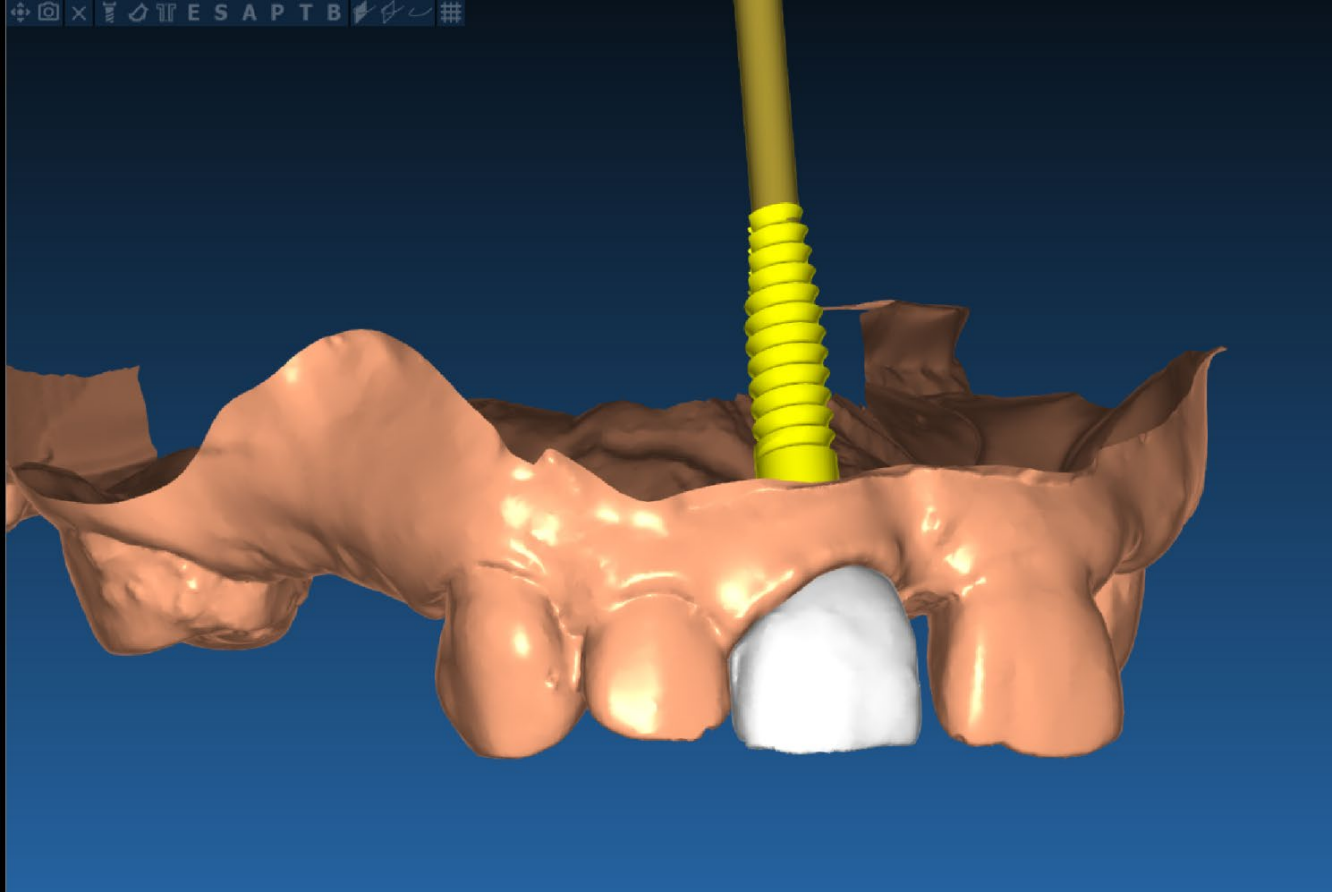
Virtual designing of the temporary prosthesis

A tooth-supported surgical guide was designed according to the prosthetically driven implant placement concept. The design of temporary restorations was carried out using (Exocad, Dental CAD software) after virtual extraction of the non-restorable anterior tooth [21].(Fig. 1).

**Fig. 2:**
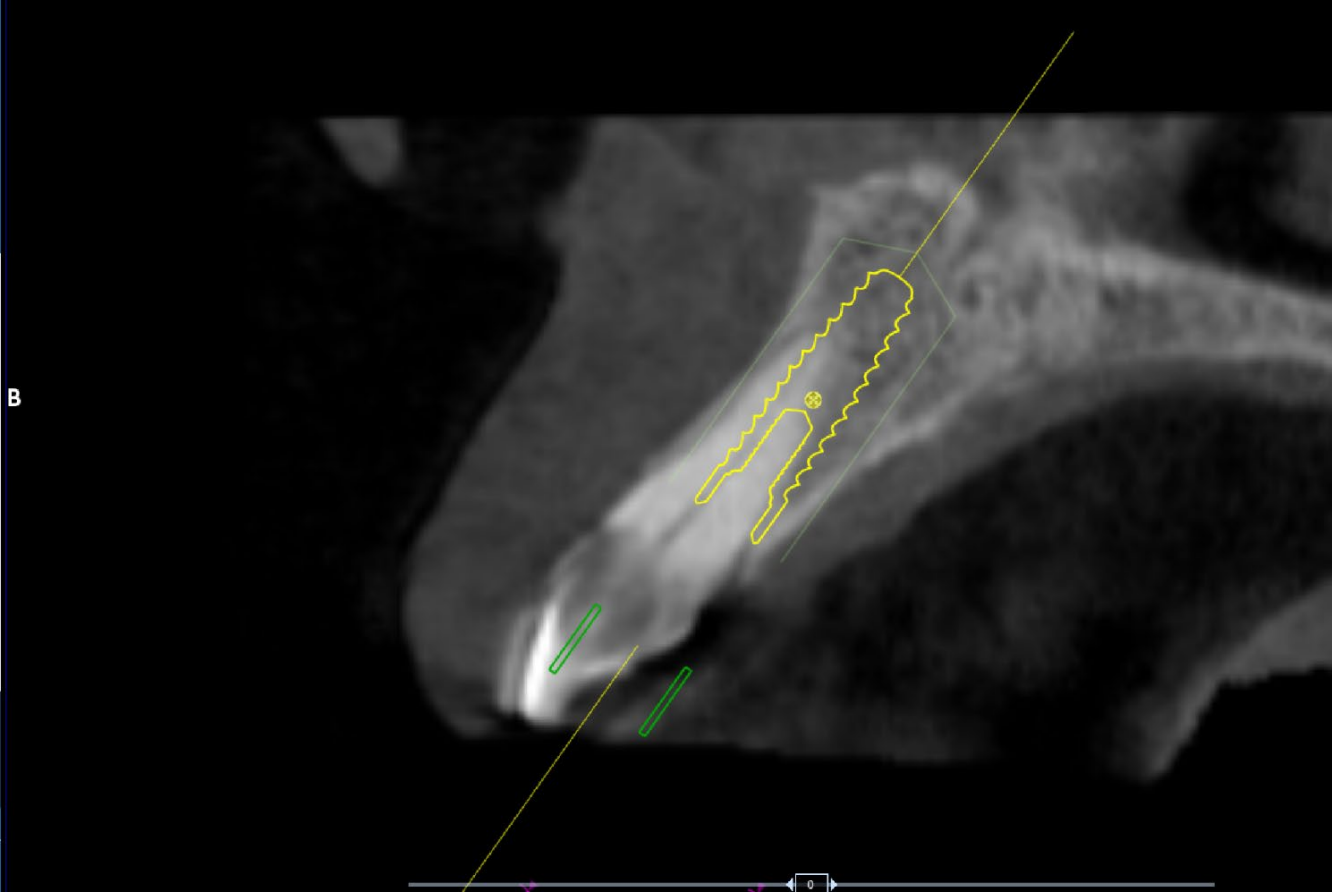
Implant planning according to the prosthetically driven implant placement protocol

**Fig. 3:**
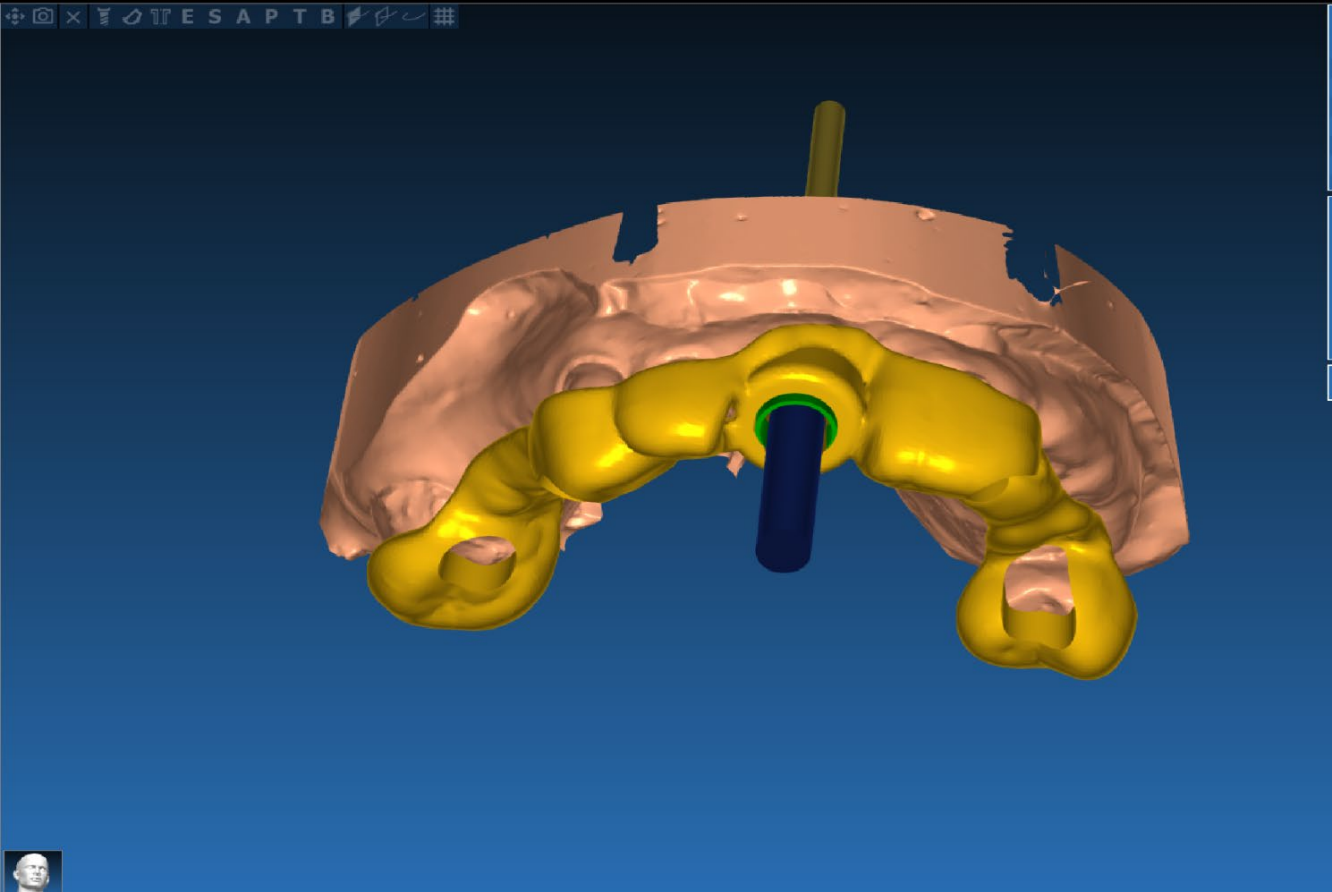
Design of the implant placement surgical guide

After superimposition of the patient’s STL and DICOM files using (Real guide 5.0 software 3DIEMME), and according to the bone width, length, and socket dimensions, the implants (implant direct Legacy; Malibu Hills Road, Calabasas Hills, CA, USA) with a diameter of (3.7–4.2 mm) and a length of (13–16 mm) were selected. (Fig. 2) The tapered implant design with progressively deep buttress threads and three cutting grooves is suggested to facilitate osseointegration. Finally, the surgical guide was generated. (Fig. 3)

The surgical guides and the temporary restorations were 3D printed using (clear resin, EPAX resin for the EPAX 3d printer) and (white porcelain-like resin, EPAX resin for the EPAX 3D printer), respectively.

### Implant placement

**Fig. 4:**
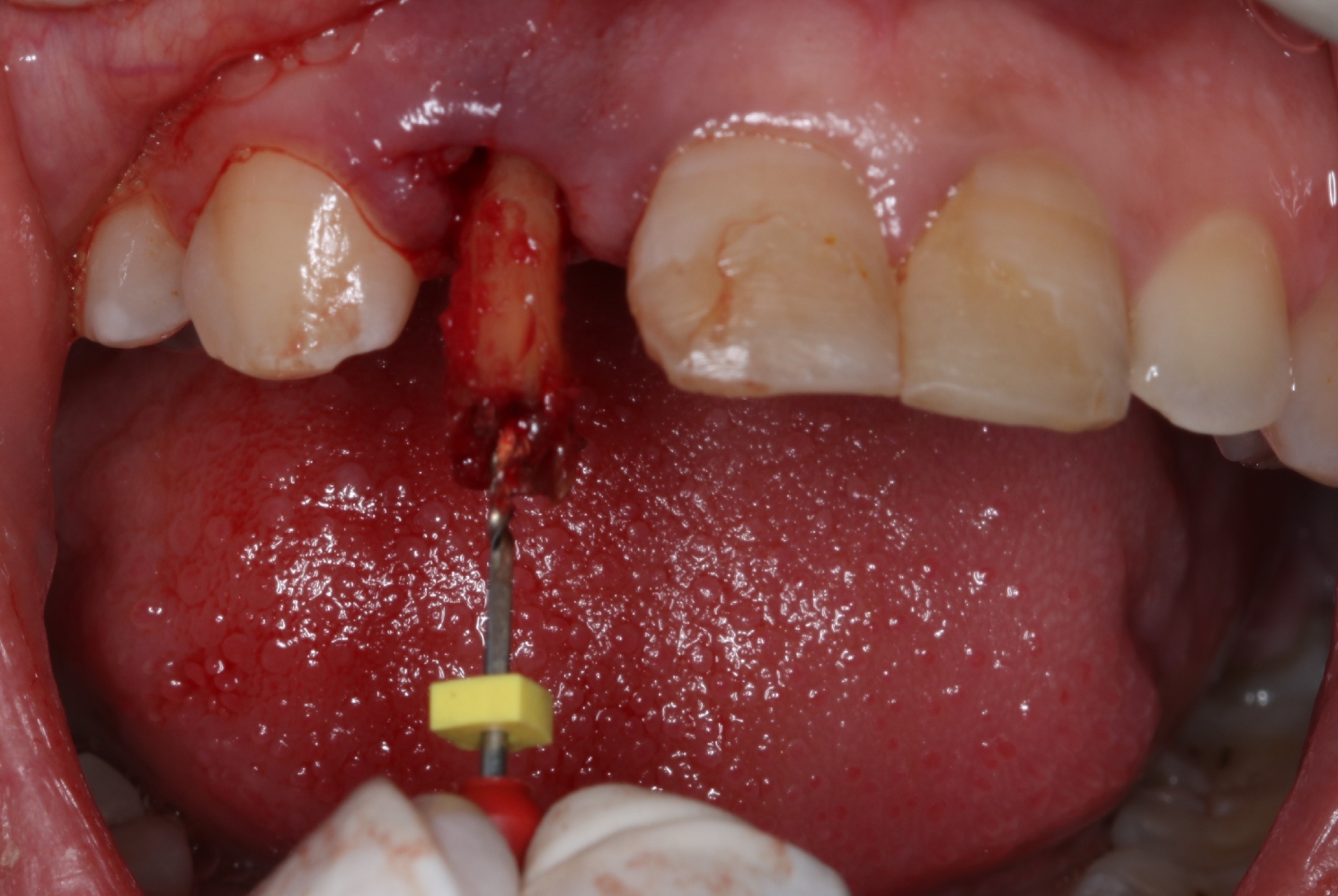
Atraumatic extraction of an endodontically treated remaining root using a large endodontic manual file

Atraumatic extraction of the tooth or remaining root using a No. 15c lancet and periotome to cut gingival fibers of periodontal ligaments was performed. In one case, we encountered a fractured root at the apical 1/3, where we carefully separated the root with a surgical bur mesiodistally (Cat eye slit technique) and removed each fragment separately. In another case, the remaining root was luxated but difficult to grip, so we removed it with a large manual file, which engaged the root canal and facilitated the root delivery. (Fig. 4) Curettage was then performed with thorough debridement using a curette and irrigation with physiologic saline to eliminate any infection, if present, before implant placement.

The implants were then placed after sequential drilling using the surgical guide. Any implants recording initial stability less than 35 Ncm weren’t subjected to immediate loading and were excluded from the study. One experienced implantologist has performed all the implant surgical procedures.

### Pickup of the 3D-printed provisional restorations

The loading of the temporary abutments was carried out over the implants. Teflon was applied around the implant into the gingival sulcus to prevent the escape of any pickup material. The pickup of the 3D provisional crowns was done with self-cured acrylic resin (Denture Base Material; Vertex-Dental B.V.).

**Fig. 5:**
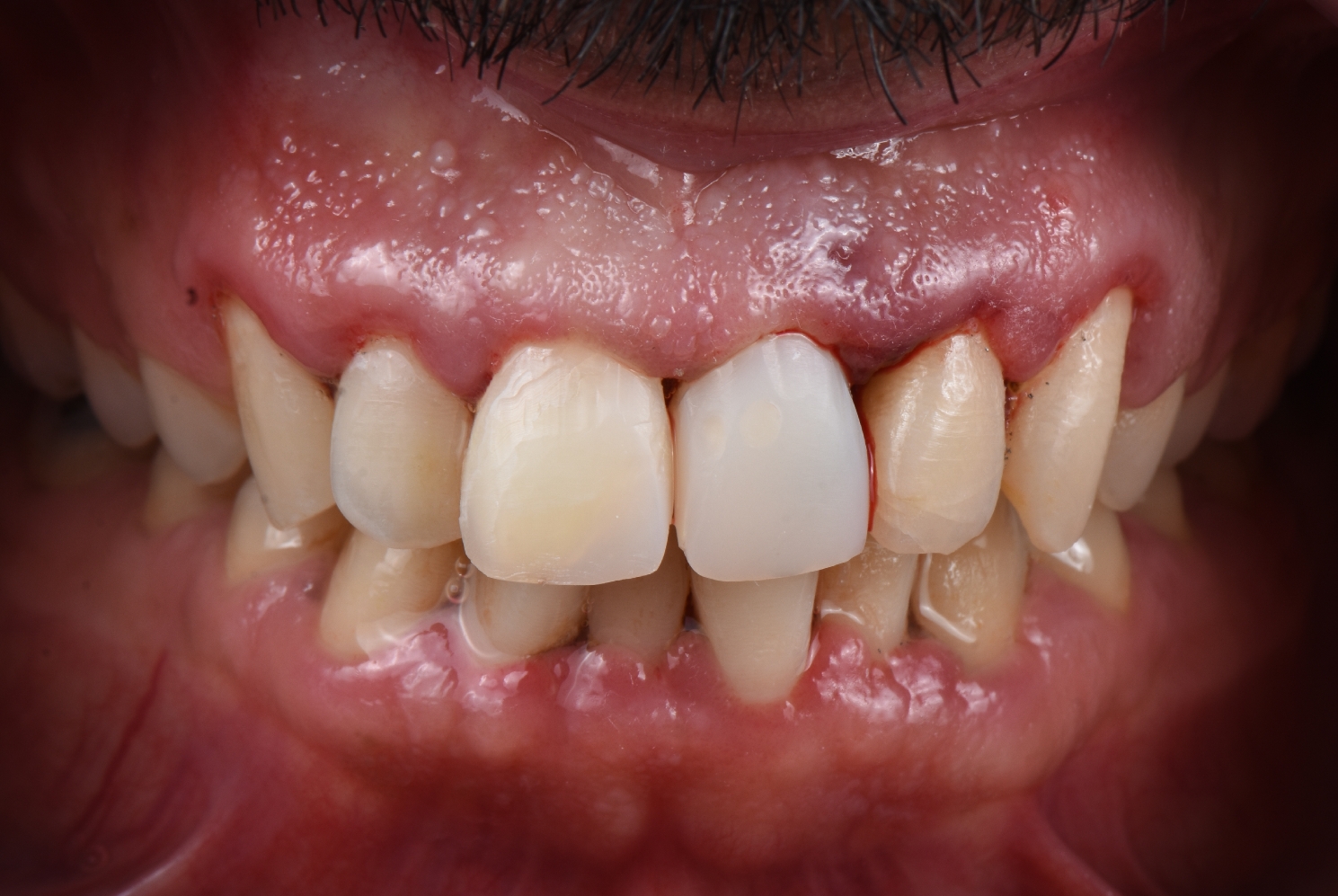
Immediate postoperative view after placing the 3D-printed temporary restoration

The provisional restorations were then finished and highly polished to achieve a smooth surface to encourage gingival formation and growth and to avoid bacterial accumulation, especially at the site of gingival contour, using rubber cups, Buff wheels, and goat hair burs at the chairside to attain an S-shaped emergence profile. (Fig. 5)

### Grouping

#### Group I. (the intervention group)

**Fig. 6:**
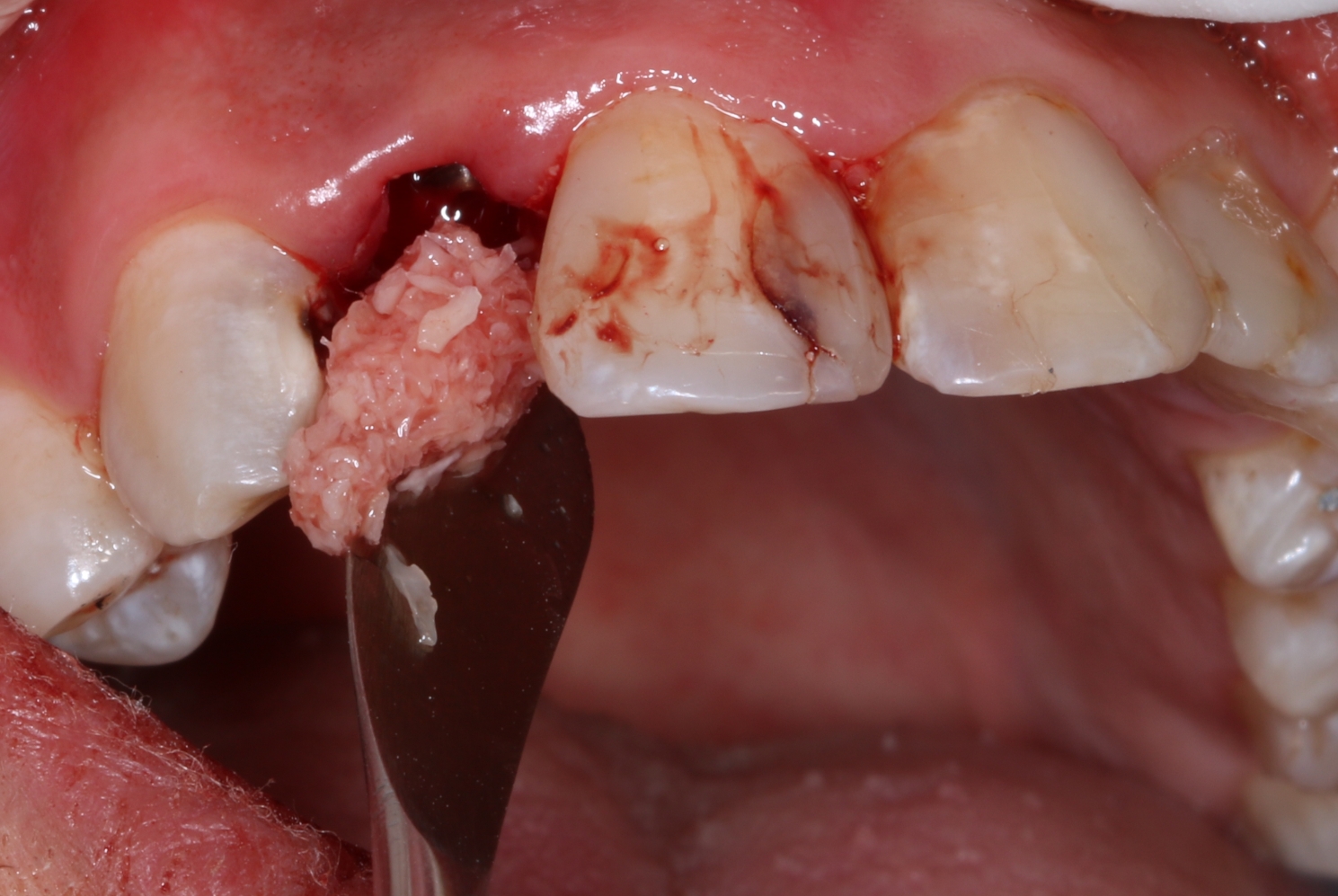
A bone mixture of 50% xenograft to 50% autogenous bone was used to graft the jumping gap in the study group

Patients were treated with immediate implant placement with a mixture of 50% Xenograft bone (Geistlich Bio-Oss®; Pharma AG, Bahnhofstrasse 40 CH -Wolhusen) and 50% autogenous bone placed in the jumping distance till the soft tissue level. The provisional restoration that was previously picked up is then screwed in place and hand-tightened. Note that autogenous bone was collected by low-speed drilling (300 rpm without coolant) from within the socket, and the bone graft material is incorporated into the tissue zone, acting as a scaffold to support the ridge contour profile and peri-implant soft tissues. (Fig. 6)

#### Group II. (the control group)

**Fig. 7:**
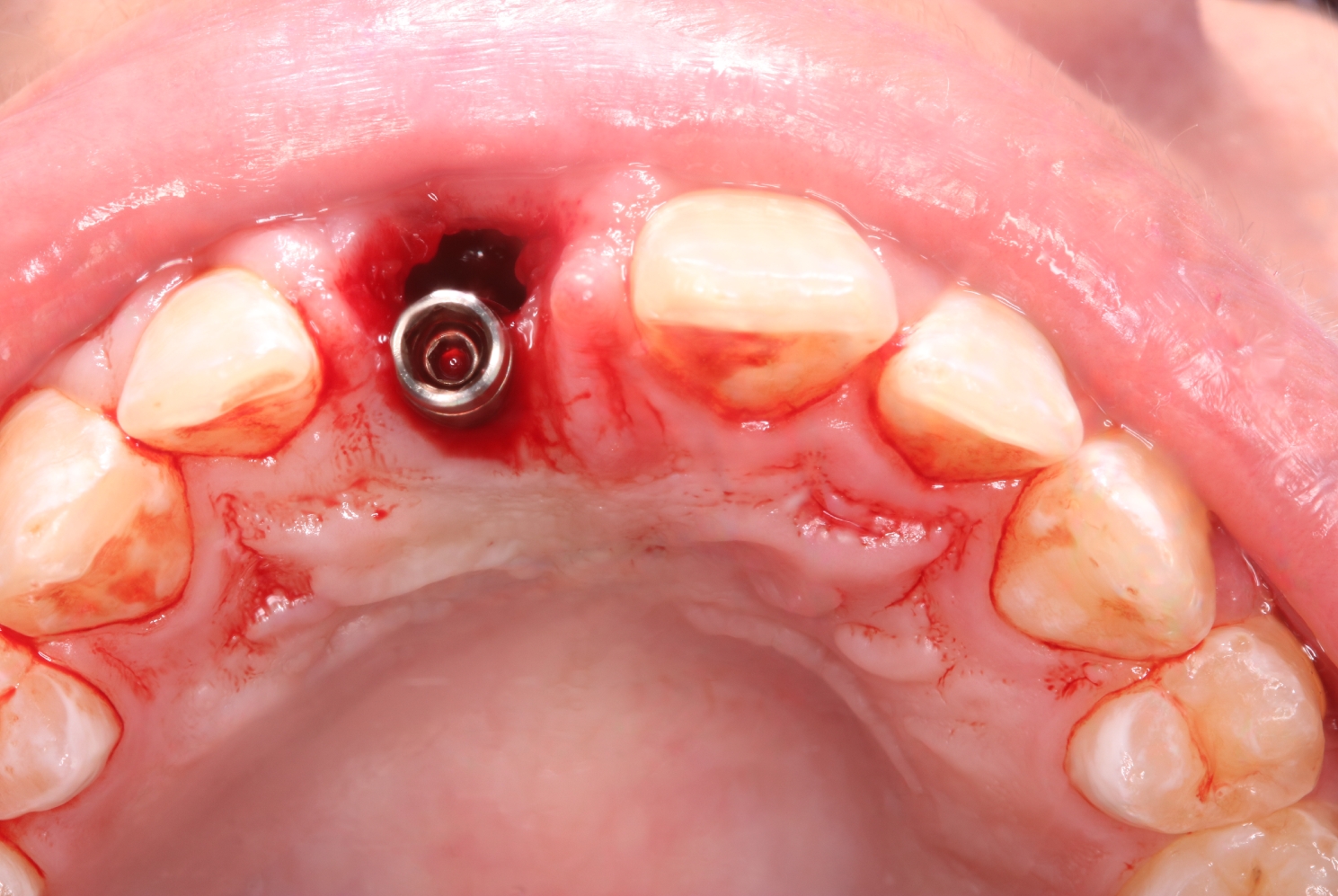
Immediate implant placement without grafting the jumping gap in the control group

Patients were treated with conventional implant placement without grafting the jumping gap, followed by hand tightening of the provisional restoration. (Fig. 7)

All patients received postoperative antibiotics orally for 3 days. Patients were instructed to follow oral hygiene measures and to use chlorhexidine 0.2% mouthwash for 2 weeks. All patients were clinically evaluated at 1 week, 2 weeks, 1 month, and 6 months postoperatively.

### Pink esthetic evaluation

The Pink Esthetic Score (PES) is based on seven variables: distal papilla, mesial papilla, soft tissue contour, soft tissue level, deficiency of the alveolar process, and soft-tissue texture and color. Each variable was assessed with a 0-1-2 score, with 0 being the worst and 2 being the best. The mesial and distal papillae were evaluated for completeness, incompleteness, or absence. All other variables were assessed in comparison to a reference tooth, i.e., the contralateral tooth [22].

### Statistical methodology

Numerical data were explored for normality by checking the distribution of data and using tests of normality (Kolmogorov-Smirnov and Shapiro-Wilk tests). The Pink Esthetic Score data showed a non-parametric distribution. Data were presented as mean, standard deviation (SD), 95% confidence interval for the mean (95% CI), median, and range values. For parametric data, the student’s t-test was used to compare the mean age values in the two groups. For non-parametric data, the Mann-Whitney U test was used to compare the two groups. Wilcoxon signed-rank test was used to study the changes by time within each group. Gender data (qualitative data) was presented as frequencies and percentages. Fisher’s Exact test was used to compare gender distributions in the two groups. The significance level was set at P ≤ 0.05. Statistical analysis was performed with IBM SPSS Statistics for Windows, Version 25.0. Armonk, NY: 1BM C0rp.

## Results

### Demographic data

Regarding baseline demographic data; there was no statistically significant difference between mean age values in the two groups. There was also no statistically significant difference between the gender distributions in the two groups. The mean and standard deviation (SD) values for age in the study group were 30.5 (9.6) years old, with 3 (25%) males and 9 (75%) females, while in the control group, the mean and standard deviation (SD) values for age were 33.8 (12.5), including 6 (50%) males and 6 (50%) females with a minimum of 20 years old and a maximum of 50 years old. Table 1.

**Table 1:** Mean, standard deviation (SD), frequencies (n), percentages (%) and results of Student’s t-test and Fisher’s Exact test for comparison between demographic data in the two groups

Demographic data	Study (n = 12)	Control (n = 12)	P. value
Age in years			0.471
Mean (SD)	30.5 (9.6)	33.8 (12.5)	
Gender			
Ma1e	3 (25%)	6 (50%)	0.4
Fema1e	9 (75%)	6 (50%)	

*: Statistically significant (p < 0.05)

NS: Statistically not significant (p ≥ 0.05)

### Pink esthetic score comparison between the two groups (inter-group comparison)

Immediately post-operatively, there was no statistically significant difference between the median PES in the two groups (P-value = 0.746, effect size = 0.13). After six months, the study group showed a statistically significantly higher median PES than the control group (P-value = 0.048, effect size = 0.859). Table 2.

**Table 2:** Descriptive statistics and results Mann-Whitney U test for comparison between PES in the two groups

Time	Study (n = 12)	Control (n = 12)	P. value	Effect size (d)
Immediate post-operative			0.746	0.13
Median (Range)	11.5 (10–14)	12 (9–14)		
Mean (SD)	11.58 (1.16)	11.75 (1.71)		
6 months				0.859
Median (Range)	13 (10–14)	11.5 (9–13)	0.048*	
Mean (SD)	12.42 (1.44)	11.17 (1.53)		

*: Statistically significant (p < 0.05)

NS: Statistically not significant (p ≥ 0.05)

### Pink esthetic score changes within each group (intra-group comparison)

**Fig. 8:**
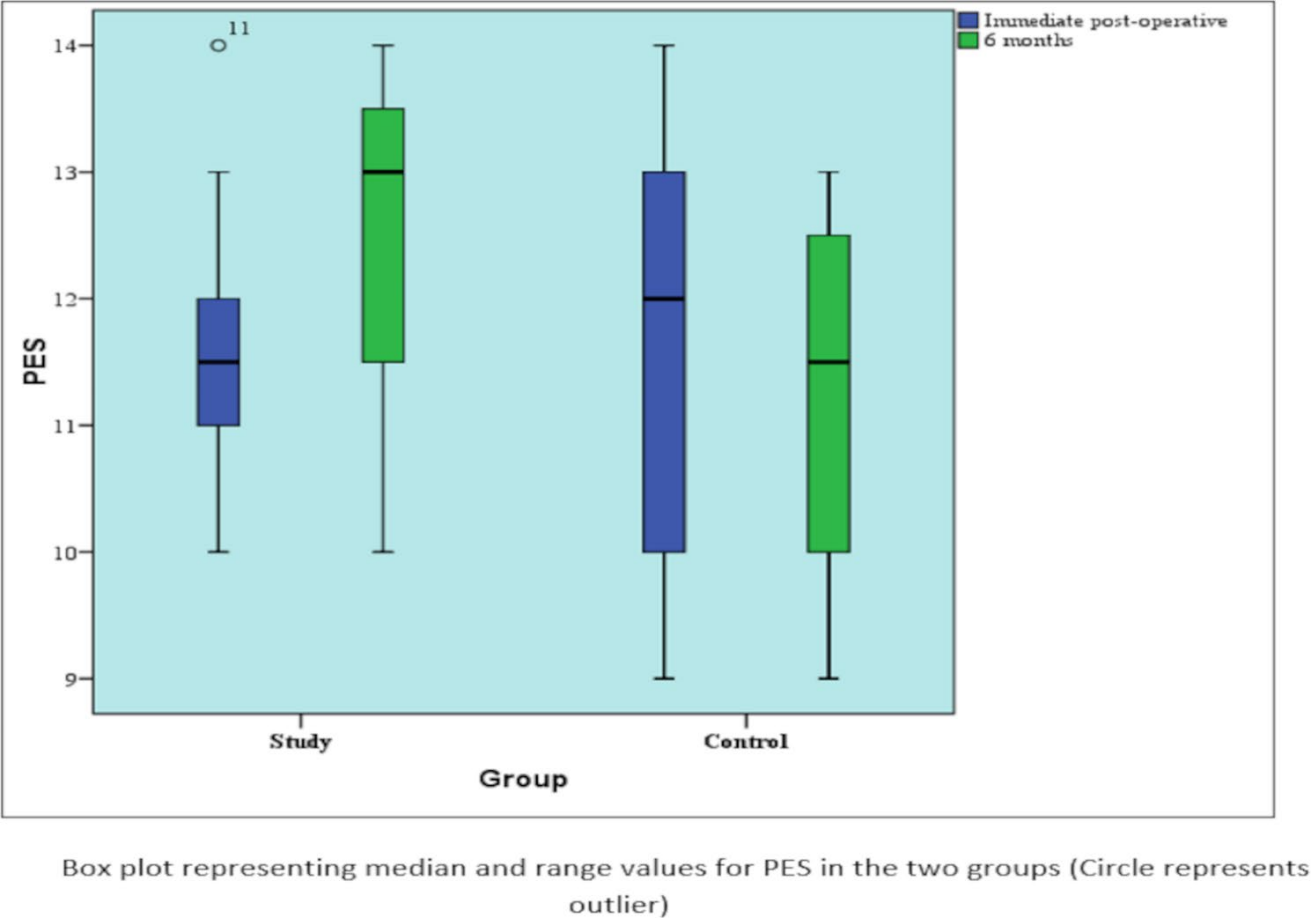
Box plot representing median and range values for Pink esthetic score in the two groups (Circle represents outlier)

In the study group, there was a statistically significant increase in PES after six months (P-value = 0.039, effect size = 0.596). In the control group, there was a statistically significant decrease in PES after six months (P-value = 0.035, effect size = 0.609). Table 3 (Fig. 8).

**Table 3:** Descriptive statistics and results of Wilcoxon signed-rank test for the changes in PES within each group

Time	Study (n = 12)	Control (n = 12)
Immediate post-operative		
Median (Range)	11.5 (10–14)	12 (9–14)
Mean (SD)	11.58 (1.16)	11.75 (1.71)
6 months		
Median (Range)	13 (10–14)	11.5 (9–13)
Mean (SD)	12.42 (1.44)	11.17 (1.53)
P. value	0.039*	0.035*
Effect size (r)	0.596	0.609

*: Statistically significant (p < 0.05)

NS: Statistically not significant (p > 0.05)

## Discussion

Many treatment protocols and materials are available to replace a natural tooth immediately after extraction, but the best protocol is unclear, especially in the esthetic zone [6, 10]. The current study was conducted to assess the pink esthetic outcome with and without grafting the jumping gap following guided immediate implant placement and a digitally fabricated temporary prosthesis. In the study group, the bone graft is added using the dual zone grafting technique, filling the bony portion and extending to the soft tissue counterpart of the temporary abutment.

The relationship between the time of implant placement and the time of extraction can be classified into delayed placement after complete bone healing, immediate-delayed placement after soft tissue healing, and immediate placement, which usually preserves the extraction socket walls [23, 24]. On the contrary, many studies agree that immediate implant placement has a high risk of mucosal recession, about 20–30%, higher than the other implant placement protocols [10, 25–28].

Mareque S. et al. reported in their systematic review that immediate implants and delayed implant placement protocols have similar results radiographically and concerning patient-reported outcomes [29].

Atraumatic tooth extraction was done to decrease the amount of labial bone resorption and maintain the gingival contour, as reported in a previous study. It was observed that atraumatic tooth extraction combined with immediate implant placement provides harmony and aesthetics to the gum line; this is attributed to the preservation of the remaining labial plate of bone during extraction and maintaining the gingiva intact without laceration [30].

When the implant is placed in a 3D position into the bone, there is usually a distance created between the outer surface of the implant fixture and the inner surface of the labial plate. This gap is referred to as the “jumping gap”. A study that identified the histologic outcome of leaving an excessive horizontal jumping gap of 4.2 mm buccolingual without bone grafting, it showed the presence of intimate bone contact until the last implant fixture thread, which is proof that an immediate implant in a socket with intact buccal bone will allow healing and osseointegration without the need for a bone graft [10, 31]. In our study, the osteotomy was drilled for immediate implant placement with palatal direction to avoid injury of the labial bone plate and to provide a room for jumping gap, the jumping distance in our study ranged from 2 to 3 mm which was determined by controlling the implant diameter.

In the study group, the jumping gap was grafted with 50% xenograft bone and 50% autogenous bone, which was collected by low-speed drilling (300 rpm without coolant) from the socket, avoiding the need for any additional surgeries. A pilot study was conducted to compare the difference between high-speed and low-speed drilling in collecting the gold standard of autogenous bone from an intra-oral site without the downside of donor site morbidity and the need for additional surgery. The samples of bone obtained by low-speed drilling showed live cells under optical microscopy, while the bone harvested at high-speed drilling didn’t show either living cells or cell growth. Also, the perfect control of drilling depth as the serrations or marks on the burs are easily visible during drilling is another advantage of the low-speed drilling protocol [32].

Xenografts play an essential role in alveolar bone preservation and can be used as a valuable tool to maintain the dimensions of the extraction socket as well as encourage osteo-conduction and space maintenance [33].

The dual-zone therapy technique means that immediate implant placement into a fresh extraction socket is performed with immediate provisionalization, and the bone grafting material is used to fill the gap until bone level and extend to the soft tissue margin. Results of this therapy show a positive outcome regarding bone healing as well as soft tissue health, giving a satisfactory esthetic outcome. This technique is recommended to attain high esthetic results, especially valuable when the gap between the implant body and the internal wall of the buccal bone is greater than 1.5 mm [34].

The implants were placed using computer-designed surgical guides according to the prosthetically driven implant protocol, which guarantees an ideal implant position. The ideal implant position and angulation would reduce the risk of facial mucosal margin recession, and the esthetic result would be favorable even in the deficient socket [5]. Many mechanical failures are due to poor prosthetic planning before implant placement such as the absence of prosthetic space or poor prosthetic design due to wrong implant distribution in addition to esthetic problems. pre-planning of the final prosthesis before implant planning contributes to the early detection and prevention of mechanical and prosthetic problems [35, 36].

The pink esthetic score (PES) assesses the soft tissue surrounding the implant at 7 points [7, 8]. The distal and mesial papilla, soft-tissue contour and level, soft-tissue texture and color, and alveolar process defect. PES may vary over time and can be a useful tool for monitoring soft tissue changes [21]. Pink Esthetic Scores of 10–12 record good esthetic results, while scores of 13 and 14 consider optimum implant esthetics [11, 37].

In our study, the temporary crown was digitally fabricated with an S-shaped emergence profile for maximum extension into the coronal soft tissue around the interim, as reported by Gluckman [38]. This interim crown should not have any contacts, either in lateral movements or maximum intercuspation. This technique of temporization allowed for soft tissue stability and high pink esthetic scores during the follow-up. The results of our study are in accordance with the study conducted by Kim TH et al., who studied the evaluation of the changes in the soft tissue following an immediate implant procedure using guided surgery in the aesthetic zone [39].

The mean results for PES for the study group were 11.58 ± 1.16 post-operatively and increased to 12.42 ± 1.44 after six months, and were 11.75 ± 1.71 post-operatively for the control group and decreased to 11.17 ± 1.53 after six months, which can be attributed to the preservation of the socket wall through the use of the bone graft, which in turn prevented the collapse of the soft tissue contour as reported previously by Noelken R et al. [40]

Based on the results in the present study, it is recommended that whenever the bony dimensions allow immediate implant with provisionalization, if it is possible, it should be the best line of treatment since it greatly enhances the PES and preserves the emergence profile with a highly finished and polished temporary crown. Satisfactory aesthetic outcomes may be achieved with immediate implants placed after the extraction of teeth in the maxillary anterior area of the dentition.

The limitations of this study are to investigate the most suitable immediate implant protocol in patients with a defective labial plate of bone and the relationship between long-term mucosal stability, and the position of the facial bone crest. Further research is needed to investigate the relationship between mucosal stability, the type of bone graft material used, and the position and thickness of the facial bone.

## Conclusions

Within the limitations of the presented study, the following could be concluded:

1-Computer-guided immediate implant placement with immediate temporalization using digitally designed infra-occlusal provisional restoration is a viable option for immediate implant replacement in the esthetic zone.

2-The socket is an ideal reservoir for the autogenous bone to thrive in.

3-Grafting the jumping distance utilizing the Dual Zone Grafting technique helps achieve a better esthetic outcome.

4-Guiding and maintaining extra bone and soft tissue volume around dental implants is the key to achieving and maintaining good soft tissue esthetics.

## Data Availability

All data generated or analyzed during the current study are included in this published article and its additional files.

## List of abbreviations

CAD-CAM: Computer-Aided Design and Computer-Aided Manufacturing
CBCT: Cone-Beam Computed Tomography
DICOM: Digital Imaging and Communication in Medicine file
PES: Pink Esthetic Score
SD: Standard deviation
SPSS: Statistical Package for Social Sciences
STL: Standard Tessellation Language file
